# Immunological studies on acupuncture and moxibustion treatment of rheumatoid arthritis: A review

**DOI:** 10.1097/MD.0000000000036875

**Published:** 2024-01-12

**Authors:** Ran Sun, Linna Wu, Yiming Sun

**Affiliations:** a Chengdu University of Traditional Chinese Medicine, Chengdu, Sichuan, China; b The First Affiliated Hospital of Tianjin University of Traditional Chinese Medicine, Tianjin, China; c National Clinical Research Center for Chinese Medicine Acupuncture and Moxibustion, Tianjin, China; d Chengdu Eighth People's Hospital (Geriatric Hospital of Chengdu Medical College), Chengdu, Sichuan, China.

**Keywords:** acupuncture, arthritis, immunity, rheumatoid

## Abstract

From the 4 perspectives of T lymphocytes, various cytokines, adenosine and “neuro-endocrine-immune” network, the researches related to the immune mechanism of acupuncture and moxibustion in the treatment of rheumatoid arthritis (RA) in recent years were summarized, and different acupuncture and moxibustion treatments were analyzed. The method has a regulatory effect on the mechanism of RA, in order to provide a reference and basis for acupuncture research on the immune mechanism of RA, and promote the further development of research in this field.

## 1. Introduction

Rheumatoid arthritis (RA) is a chronic, systemic autoimmune disease characterized by synovial inflammation of the joints. Its main pathological features include synovial inflammatory exudation, cell infiltration, pannus formation, progressive erosion, and destruction of articular cartilage. The clinical manifestations are pain, swelling, and deformity of multiple joints, which lead to the impairment or even loss of joint mobility.^[1,2]^ Approximately 25% of patients develop disability in life and work within 3 years after the diagnosis of RA, and up to 35% develop work difficulties within 10 years.^[3]^ The incidence of RA in China is about 0.42%, which is slightly lower than the global incidence of 0.5% to 1%, but it has shown an increasing trend year by year in recent years.^[1,4]^

At present, the pathogenesis of RA is still inconclusive, and there is no specific treatment in clinical treatment.^[5]^ At present, leflunomide and nonsteroidal anti-inflammatory drugs are mainly used for symptomatic treatment, but adverse reactions such as nausea and vomiting, impaired liver and kidney function after taking drugs, and side effects such as drug dependence after long-term use have gradually attracted attention.^[6]^ Approximately 30% of patients with RA seek complementary and alternative therapies, which include acupuncture.^[7]^ Studies have shown that acupuncture, moxibustion, and electroacupuncture all have good curative effects on RA without obvious adverse reactions.^[8]^ Studies have found that acupuncture and moxibustion participate in immune response, regulate immune links including immune cells and immune factors, exert immune modulatory functions such as analgesia, anti-inflammation, anti-infection, and antitumor, and play a positive role in the prevention and treatment of allergic diseases and infectious diseases.^[9]^

In this paper, the researches related to the immune mechanism of acupuncture and moxibustion in the treatment of RA are summarized from the 4 perspectives of T lymphocytes, a variety of cytokines, adenosine and “neuron-endocrine-immune” network in recent years, so as to provide more reference and basis for the further development of research related to the immune regulation mechanism of acupuncture and moxibustion.

## 2. T lymphocyte regulation

The occurrence of RA is related to the activation of cellular immunity, and the functional status of T-lymphocytes can reflect the immune function of the body. The dysfunction of T lymphocytes can lead to a decrease in the body’s defense resistance, which brings resistance to the recovery of RA patients.^[10]^ CD4^+^T lymphocytes (cluster of differentiation 4, CD4) can differentiate into different T cell subsets when stimulated by antigen-presenting cells, among them, there are T helper 1 cell, T helper 2 cell, T helper 17 cell. Th17 and other related cytokines are dominant,^[11]^ and the imbalance of Th17/Treg and Th1/Th2 cytokines is considered to be one of the mechanisms causing the pathogenesis of RA, and the restoration of their proportion balance is a potential mechanism of action for RA treatment.^[12,13]^ Zhu J et al found that electroacupuncture stimulation could significantly increase CD4^+^FOXP3^+^ (forkhead/winged helix nuclear transcription factor 3) expression of Treg cells, which decreases CD4^+^IL17^+^ (interleukin-17) expression frequency of Th17 cells, reestablishing the Th17/Treg cell balance.^[14]^ Triptolide, human serum albumin nanoparticles and cyclopentyl adenosine were injected into Zusanli (ST36). The results showed that acupoint injection of CCPA had specific analgesic effect, inhibited pro-inflammatory cytokines and increased CD4^+^CD25^+^Expression of FOXP3 + Treg cells, significantly reduced CD4^+^IL17A^+^Th17 cells, which balance Th17/Treg, inhibit the further progression of RA course.^[15]^ The regulation of T lymphocytes, improvement of Th1/Th2 cell differentiation imbalance, and alleviation of knee joint inflammation may be achieved by stimulating Treg cells to reduce the content and gene expression of Th1 inflammatory cytokines in serum, and increase the content of Th2 marker cytokines and the expression level of Foxp3.^[16]^ This study provides an important basis for further research on the molecular signaling mechanism of acupuncture activation.

## 3. Regulation of cytokines

The destruction of the body’s immune balance is directly related to the occurrence of RA. Cytokines are important mediators of inflammation and joint damage in RA, which aggravate joint inflammation and hyperalgesia in RA patients. These cytokines include tumor necrosis factor-α (TNF-α) and interleukin-1β (IL-1β). interleukin-6 (IL-6), matrix metalloproteinase, vascular cell adhesion molecule-1 (VCAM-1), vascular endothelial growth factor (VEGF), and others.^[17,18]^ The release of chemokines in RA patients is often caused by inflammatory cytokines, which induces a large number of inflammatory cells to migrate from the peripheral blood to the synovial membrane of the joint and destroy it, and participates in the activation and proliferation of T lymphocytes and the formation of pannus. interleukin-1 is one of the most important inflammatory cytokines.^[19]^ Gusmao J et al found that EA treatment could significantly reduce the serum levels of IL-6 and IL-17, reduce inflammatory cell infiltration, inhibit the aggregation of osteoclasts, and reduce inflammatory parameters in RA rats with periodontitis.^[20]^ Zhang Rui et al found that electroacupuncture can improve the infiltration of inflammatory cells in collagen-induced arthritis (CIA) rats and inhibit the positive expression of IL-1β in synovial membrane and bone tissue of CIA rats. It reduces the content of intercellular cell adhesion molecule-1, TNF-α and IL-1β in serum and blocks the necessary links of inflammatory response in CIA rats.^[21]^ It can also inhibit the overexpression of VEGF protein, IL-17, and IL-23 in knee tissues, block the IL-23/IL-17 axis, inhibit local angiogenesis, and delay the destruction around the joint.^[22]^ Similar to EA treatment, fire needling treatment reduced the serum levels of TNF- and anti-cyclic citrullinated peptides antibody, and alleviated local inflammatory cell infiltration in RA rats. Local synovial tissue destruction and bone damage are slowed down.^[23]^

An infrared smart laser device system did noninvasive remote laser acupuncture points stimulation; this allowed the clinicians to adjust the laser device settings and parameters remotely; in the current environmental conditions of the COVID-19 pandemic, the advantages of noninvasive remote laser treatment options are significant.^[24]^ After laser acupuncture treatment, the C-reactive protein, IL-6 and malondialdehyde oxidation markers of the patients were significantly lower than those before treatment. ATP (adenosine triphosphate), SOD (superoxide dismutase), GR (glutathione reductase). The antioxidant markers glutathione reductase and glutathione (reduced glutathione, glutathione) were significantly increased after treatment (*P* < .05), indicating that laser acupuncture had significant anti-inflammatory and anti-oxidative effects.^[25,26]^ The levels of COMP (Cartilage Oligomeric Matrix Protein) and COII (cytochrome c oxidase subunit II) were increased. It can effectively delay cartilage degradation and reduce the degree of destruction of articular cartilage.^[27]^

Fu Wen et al found that both acupuncture and moxibustion could effectively reduce the expression of IL-17 in the serum of RA model rats and prevent the synergistic effect of synovial cells with IL-1 and TNF-α activation. By reducing the content of MMP and other pro-inflammatory factors to maintain the balance of MMP-9/TIMP-1 (tissue inhibitor of matrix metalloproteinases-1), protect articular cartilage and bone and joint. Thereby controlling the occurrence of synovial inflammation.^[28]^ The levels of IL-17 and TNF- in serum of rats with collagen-induced arthritis after moxibustion treatment are decreased, which blocks the inflammatory response and has a positive effect on Th17/Treg cell balance.^[29]^ Some studies also believe that moxibustion plays an anti-inflammatory and analgesic therapeutic effect by reducing the expression of nuclear factor kappa-B (Nf-κb) and VEGF, preventing the further growth of pathological blood vessels and reducing the formation probability of synovial pannus, thereby relieving the local inflammatory symptoms of joints.^[30,31]^

The combined application of moxibustion therapy and oral leflunomide tablets or methotrexate tablets has additive effect, which can better improve the clinical symptoms of RA patients and significantly reduce the expression levels of serum hypoxia inducible factor-1 and VEGF (*P* < .05).^[32]^ Wang Lihua et al, after treating “Zusanli,” “Xuanzhong,” and “Shenshu” with warm acupuncture, found that the expression of VCAM-1 protein in the synovial tissue of the model group was higher, while the expression of VCAM-1 protein in the acupuncture group and the warm acupuncture group was lower than that in the model group, and the effect of the warm acupuncture group was more obvious than that of the acupuncture group. This experimental result suggests that warm acupuncture can mediate the production of immunoglobulin and interfere with the expression of related inflammatory factors.^[33]^ Similarly, some clinical studies have found that warm acupuncture can improve the clinical joint dysfunction of patients and reduce the content of CRP, IL-1, IL-6, and TNF- in peripheral blood, which is an effective method for the treatment of active RA.^[34]^ The effects of different moxibustion techniques on therapeutic effects should also be considered in clinical treatment. Zhong YM et al found that suspension moxibustion was significantly better than wheat-grain moxibustion in improving the levels of inflammatory cytokines when the subjects of study were RA animal models by comparing moxibustion methods in various studies.^[35]^ The regulatory effect of acupuncture and moxibustion on cytokines in RA patients/animals has been confirmed from multiple perspectives.

## 4. Adenosine receptor modulation

Adenosine is an important endogenous nucleoside, which is distributed in various parts of the human body, especially in the central nervous system. It is involved in physiological activities such as sleep, respiration, heart rate and nerve transmission by binding to different receptors, and plays a role in regulating vasodilation, lowering blood pressure and heart rate. In the inflammatory response, adenosine plays an anti-inflammatory role, relieving pain and inhibiting the activation of inflammatory cells mainly through adenosine receptors (ARs) on the surface of cell membrane.^[36]^ Activation of ARs is able to regulate the release of inflammatory cytokines, and a large number of studies have shown that modulation of adenosine signaling is a potential strategy for the treatment of RA.^[37]^ Han Jing et al studied the expression of adenosine A1 receptor (ADORA1) in the hypothalamus and spinal cord of adjuvant arthritis (AA) rats. Various inflammatory stimuli in RA state inhibit the activity of central ADORA1 receptors, while electroacupuncture treatment can significantly improve this situation and promote the combination of endogenous adenosine or adenosine A1 receptor agonists with this receptor to mediate the information transmission of inflammatory pain, thus effectively playing an analgesic and anti-inflammatory effect.^[38]^

## 5. Regulation of “neuron-endocrine-immune” network

With the deepening of related research, the concept of “neuro-endocrine-immune network” (NEI) cytokine network has attracted more and more attention in the pathogenesis of RA.NEI network means that the nervous system, endocrine system, and immune system not only regulate the body independently, but also exchange and share information through neuropeptides, endocrine and other cell signaling molecules and their receptors. Disorders of the overall function of NEI network can lead to the occurrence of various diseases.^[39]^ In the process of RA, a variety of intersecting pathophysiological systems such as autoimmune inflammation and endocrine disorders jointly form an intricate NEI network.^[40]^ Yang F et al found that the foot swelling of AA rats was reduced after manual acupuncture of ST36, the levels of TNF-α protein and other pro-inflammatory factors in the ankle joint were down-regulated, and the cellular immune network in the inflammatory site was enhanced (the cell network density was 230.00 on the first day of acupuncture; Cellular network densities at days 7 and 15 were 40.82 and 86.76, respectively), demonstrating that acupuncture can increase the strength of immune effects on target organs, and at the same time promote the differentiation of macrophages in the joint site to the M2 phenotype, and secrete cytokines that inhibit inflammation and promote tissue repair.^[41]^ Ding Shasha et al observed 32 common signaling molecules of NEI network in the serum of 131 Wistar rats. The results showed that, compared with the model group, the secretory cells in the acupuncture group were mainly in the hypothalamic pituitary neurons, the transmitter cells were mainly monocytes and macrophages and the target cells were mainly brain neurons and spinal cord neurons, indicating that acupuncture can activate “NEI” cells, and acupuncture at Zusanli point can significantly increase the thermal radiation pain threshold and reduce inflammatory response in AA rats with inflammatory pain, and its analgesic and anti-inflammatory effects on AA rats are achieved by regulating intercellular communication of NEI network.^[42]^

## 6. Discussion

In conclusion, RA has an extremely complex pathological process, and various systems interact with each other to jointly affect the development of RA. In recent years, the number of studies on the mechanism of acupuncture and moxibustion in the treatment of RA from the perspective of cytokines has gradually increased, reflecting that acupuncture, and moxibustion can inhibit the development of RA by affecting a variety of cytokines, improve the clinical symptoms of RA patients and improve their quality of life. T lymphocytes and inflammatory cytokines play an important role in the occurrence and development of RA. Firstly, acupuncture can restore the function of T lymphocyte population and reconstruct the balance of Th1/Th2, Th17/Treg and other cytokines, thereby inhibiting the development of RA. Acupuncture can also reduce the expression of IL-1, IL-17, IL-23, TNF-α and other related inflammatory cytokines, prevent the activation and synergistic effect of synovial cells with IL-1 and TNF-α, reduce the infiltration of inflammatory cells to regulate the body’s immune balance, prevent the formation of local synovial pannus, delay the destruction of joint synovial tissue, and protect the articular cartilage and bone joint. Adenosine, as an important endogenous nucleoside, participates in the related inflammatory response through adenosine receptors on the surface of cell membrane and regulates the release of inflammatory cytokines. Acupuncture and moxibustion can significantly enhance the activity of ADORA1 receptors, inhibit the information transmission of inflammatory pain, and play an analgesic and anti-inflammatory role. On the basis of the regulation of nervous system, endocrine system and immune system, NEI network emphasizes the sharing of cell signaling molecules such as neuropeptides and endocrine system. After acupuncture, the strength of immune effect is enhanced, and the cytokines related to inflammation inhibition and tissue repair are improved, so as to achieve labor pain and anti-inflammatory effects.

Because current studies are mostly limited to the exploration of a single target and lack standardized treatment regimens,^[43]^ the overall regulation of acupuncture and moxibustion on the human body and the interaction between various cytokines cannot be fully reflected. Therefore, the author believes that the NEI network as the core of multi-system collaboration research may be one of the main research directions in the future. As an important component of traditional medicine, acupuncture and moxibustion therapy emphasizes that “every needling method must first be based on the mind,” that is, “treating the mind” and “keeping the mind.” The doctor needs to treat the patient with a holistic idea, which is reflected in the selection of acupoints and the operation of acupuncture and moxibustion. The regulation of acupuncture and moxibustion on the body is not single, and the treatment mechanism of RA is complex and diverse. Different acupuncture and moxibustion treatment methods have different therapeutic advantages. If the integrity of the human body and acupuncture and moxibustion therapy is ignored, only centralized and independent research on one system cannot interpret the internal relationship of the body’s various network systems at a macroscopic level.

Although the immune mechanism of acupuncture and moxibustion therapy is still not completely clear, we hope to deepen the research through the development of modern medicine and the progress of science and technology, so as to play the unique advantages of acupuncture and moxibustion to relieve the pain of RA patients, and also lay the foundation for the modernization of traditional medicine.

## Author contributions

**Writing – original draft:** Ran Sun.

**Writing – review & editing:** Ran Sun, Wu Linna, Yiming Sun.
